# The Investigation of Persistent Diarrhoea

**Published:** 1958-10

**Authors:** John Naish

**Affiliations:** Consultant Physician, Bristol Clinical Area


### THE INVESTIGATION OF PERSISTENT DIARRHOEA

BY

JOHN NAISH, M.D., F.R.C.P.

*Consultant Physician, Bristol Clinical Area*

Diarrhoea is the passage of loose stools so frequently as to cause annoyance. To some patients, four bowel actions a day is an intolerable nuisance, some are much more stoical, while others complain freely when the inconvenience is less. The severity of pain, urgency and tenesmus accompanying the diarrhoea often determines whether a patient seeks medical help or not. By persistent diarrhoea is meant the occurrence of frequent loose stools, continually or intermittently for a period of over three weeks.

It is true that many patients with chronic diarrhoea begin their illness abruptly, but the problem of acute flux itself is beyond the scope of this paper. Whereas in acute cases the emphasis is on the search for and treatment of an infective agent, together with the palliation of symptoms and treatment of dehydration, in the case of persistent diarrhoea the search for a cause has spread far beyond the confines of infection. Indeed, in temperate climates an infective cause is the exception rather than the rule. Yet the almost automatic action on the part of many doctors faced with a case of persistent diarrhoea is to obtain a specimen of faeces and send it many miles to a public health laboratory with "? *organisms*" on the request card. The patient's difficulties with the ice cream carton provided are matched by those of the bacteriologist at the other end, who naturally finding plenty of organisms has to report in such terms as "*No significant departure from the normal bowel flora*". In circumstances like these it is well to ask what precisely we are looking for, and I suppose the answer would be "typhoid and paratyphoid, and things like that". It is true that in infants and children, cases of paratyphoid fever may occur with persistent diarrhoea as a main symptom, but in the adult, diarrhoea if it occurs at all is completely overshadowed by the feverish and constitutional symptoms. It is true also that in the very old or debilitated, chronic Sonne Dysentery or other salmonella infections may cause persistent diarrhoea. In these cases, especially if the illness is accompanied by fever, the bacteriological search for non-lactose fermenting organisms by culture is worthwhile.

Where the patient has been abroad and chronic amoebic dysentery or lambliasis are suspected the examination of old stale specimens of faeces is completely useless. If these diseases are suspected the faeces should be taken straight from the rectum to the microscope slide, preferably after a sigmoidoscopic examination, but failing that a fresh stool should be taken straight to the laboratory in the bed pan, and the search for parasites begun at once. Worms do not give rise to diarrhoea in this country.

Many man-hours and money are wasted on fruitless pathological examinations of faeces. It is a form of long distance investigation that could well be dispensed with. In fact, the diagnosis of continuing diarrhoea is much more direct, though it is not simple. What is required is a finger stall or glove, a good illuminated proctoscope or small sigmoidoscope, some powders or tablets for testing for occult blood, and a syringe to withdraw some blood for the estimation of haemoglobin and E.S.R. at the nearest laboratory. Other investigations may be necessary, but one can get a long way with the simple procedures.

Table I gives a list of the commoner disorders which give rise to persistent diarrhoea, and these are arranged in main groups according to the basic mechanism and pathology. Table II gives the expected findings in each group with each of the main investigatory procedures.

TABLE I

CLASSIFICATION OF CAUSES OF PERSISTENT DIARRHOEA

| Main Groups | Varieties |
|---|---|
| Nervous and functional .. | "Lienteric". "irritable colon syndrome". "Mucous colitis." Episodic with vomiting. Associated with general diseases. |
| Malabsorbtion .. .. | "Sprue". Coeliac Disease. Idiopathic steatorrhoea. Pancreatitis. Loop syndrome. After gastrectomy. Hepatogenous. |
| Inflammatory .. .. | Ulcerative colitis. Proctitis. Ileitis. Ileo-colitis. Diverticulitis. |
| Infective .. .. | Amoebic dysentery. Chronic dysentery. Lambliasis. |
| Neoplastic.. .. .. | Carcinoma of colon (commonly caecum or rectum). Rare tumours of the small intestine. |

*Rectal examination*—one cannot overstress the importance of making a digital rectal examination in all patients with chronic diarrhoea. The fact that carcinoma of the colon is one of the most operable of all carcinomas and that 30 per cent of colonic carcinomas occur in the rectum should stimulate us to make this essential if unpleasant examination. Small finger stalls which are easily disposable are, outside Hospitals, more practicable than the flanged, heavier type. If carcinoma is suspected the finger should explore as high as possible, and bimanual examination be attempted.

*Occult blood tests*—there are now a variety of tablets and powders which when dissolved in glacial acetic acid and poured onto a blood-containing faecal smear on paper or glass give rise to a dark blue or green colouration. Gregerson's powder is no longer obtainable because the makers will not handle Benzidine which has been proved to be carcinogenetic in animals. An alternative powder is made up of Barium Peroxide 0.2 g. and Ortho Tolidine 0.025 g. "Hematest" tablets can also be used for testing a faecal smear by placing the tablet on the faecal smear and moistening the tablet and smear with two drops of water. A strongly positive occult blood test is nearly always significant of intestinal bleeding, though it must be remembered that the bleeding can be from the nose, the gums, or the anal canal. The test is not positive in the presence of iron, but intestinal hurry may cause haemoglobin rich animal meat fibres to appear in the stool and give a weakly positive (green) reaction.

Two or three successive positive tests are more significant than a single positive test, but the finding of a negative test is strong evidence against carcinoma or ulcerative colitis. In this way the rectal examination and subsequent occult blood test of a faecal smear obtained from the examining finger stall or glove provide a useful "screening" procedure, negative findings tending to promote a sense of security and positive finding being regarded as a call for immediate further investigation.

*Proctoscopy and sigmoidoscopy*—in out-patient practice the rectum only is inspected. A simple small, proximally lighted sigmoidoscope is the best instrument. The presence of faeces may prevent a careful examination of the upper rectum, but they rarely conceal the appearance of the rectal mucosa. In 95 per cent of cases of ulcerative colitis, the mucosa has a very typical reddened, granular appearance. The veins so visible in normal mucosa, cannot be seen in cases of ulcerative colitis. The mucosa also bleeds easily and ulcers may be seen, though the last finding is not essential for a diagnosis.

Amoebic ulcers are small irregular and pitted, surrounded by a rim of red mucosa, but beyond this all is normal.

The appearances of malignancy vary: there is rarely any doubt as to the diagnosis in this country, though infective granulomas may be a source of difficulty in the tropics.

*Stool appearance*—the patients description of the frequency, and appearance of the stools is valuable evidence, but the appearance of the stool at sigmoidoscopy, or as

TABLE II

THE FINDINGS IN THE MAIN GROUPS OF CAUSES OF PERSISTENT DIARRHOEA

| Main Groups | P.R. | Occult blood | Sigmoidoscopy | Stool appearance | E.S.R. | Hb. | Barium enema | Daily fat excretion in stools |
|---|---|---|---|---|---|---|---|---|
| Nervous and functional .. | Nil | — | Nil (or excess mucous) | Varies | Normal | Normal | ? spasm | Normal |
| Malabsorbtion .. .. | Nil | — | Nil (mild hyperaemia sometimes) | Fatty | Normal | Low | Normal | Over 5 gms. |
| Inflammatory .. .. | ? granular mucosa | + | Diagnostic in 95 per cent of ulcerative colitis and proctitis | Offensive blood ± | Much raised | Low | Usually abnormal | Usually normal |
| Infective .. .. | Nil | ± | ? amoebic ulcers | Offensive blood ± | ? | ? | May be normal | Normal |
| Neoplastic.. .. .. | ? mass | + | ? mass or blood from above | Offensive blood ± | Raised | Low | Filling defect seen | Normal |

inspected in the bed pan may give the clue to diagnosis. The stool in the sprue—like malabsorbtion syndromes is bulky, pale and greasy in appearance. Often it is frothy. The presence of mucus is not to be taken as alarming, for this is a common finding in many functional disorders of the intestine. The presence of visible blood coming from above the anal canal is of immense significance.

*E.S.R. and haemoglobin*—these are rarely normal in ulcerative colitis and Crohn's Disease. In malabsorbtion states, the haemoglobin may be low and the E.S.R. normal. The same applies to carcinoma of the colon. These tests are simple to perform in a laboratory, or in the clinic, and as with the occult blood test they are most useful as a "screening" or preliminary procedure.

*Barium enema*—this is often the only way of demonstrating disease of the caecum or terminal ileum whether it be inflammatory or neoplastic. In ulcerative colitis, a barium enema tells something of the extent of the disease and the severity of mucosal involvement. As a routine procedure for the exclusion of carcinoma of the colon it is not recommended unless the limitations of the method are fully understood. A negative report does not mean that a carcinoma is not present, it merely means that with the relatively crude methods available, a carcinomatous filling defect has not been seen. Faeces still present after bowel lavage are the greatest source of difficulty in interpretation. In general, a barium enema should not be done until all the other investigations have been made and evaluated, and the report should be placed in perspective.

*Enfoliative Cytology*—no really satisfactory technique for the diagnosis of colonic carcinoma using this method has been devised and, though many examinations are made in America, it is not considered a practical proposition over here.

*Assessment of probabilities*—in the end one comes back to clinical judgement in all problems of diagnosis, and this is particularly true when considering a case of persistent diarrhoea. So far the mechanical, chemical and haematological aids to diagnosis has been stressed with a rather different clinical approach to this problem because ordinary "routine" medical examination yields so few clues in this type of case. Nevertheless, at the end of all investigations, there is often a need to reach an opinion based on conflicting evidence; or on insufficient understanding of the pathological and physiological factors controlling intestinal function. So little is known about the mechanism of chronic diarrhoea: why, for example, one patient with ulcerative colitis will pass twenty stools a day, whereas another with the disease apparently of the same extent and severity, passes two stools a day. And so little is known about the mechanisms behind so many of the functional bowel disorders—those distressing conditions where the patients seem to have lost all the natural rhythym of their intestinal function. Some cases of diarrhoea last three to six months, and then clear spontaneously for no known reason. Sometimes in these cases the stools may appear fatty and actually contain excess fat, and it is clear that the disorder is of the small intestine or pancreas, or both; it seems, however, to be self limiting. In other rather similar cases, the rectal mucosa may appear hyperaemic, and one is inclined to think in terms of an allergic basis. However there is no doubt that it is to the patients' benefit if an early diagnosis is made of ulcerative colitis, or carcinoma of the colon, and that appropriate treatment can alleviate the troubles of those with malabsorbtion syndromes, or of those with functional diarrhoea.

"*Functional Diarrhoea*"—this subject requires a special note because the diagnosis is based not only on the negative results of investigations, but also on the pattern of symptoms and the patient's general history, physical constitution and behaviour.

Certain symptom complexes occur more frequently than others. The so-called Lienteric Diarrhoea which is an exaccerbation of the normal gastro-colic reflex, is rarely a cause for concern, but may sometimes cause alarm to an anxious parent, or an anxious patient. Reassurance and explanation do much good.

*The irritable colon syndrome—alias* spastic colon, is characterised by the loss of any regular bowel habit. Phases of constipation are often succeeded by violent diarrhoea and sometimes the latter becomes a habit. Small hard, or normal stools, surrounded by copious mucous are often passed. One variant is the badly named "*mucous colitis*" where an irritable rectum constantly desires to empty itself of small quantities of mucous which has been produced by over-active, over-stimulated goblet cells.

There are also sufferers from an over-sensitive rectum, whose urgent defaecation becomes a habit whenever they have to keep an appointment or leave the house. The trouble becomes a habit and an obsession, interfering with and modifying their lives. It is often associated with pruritus, strangury and genital symptoms. The basis may be emotional, but one sometimes feels that some of the sufferers are unlucky to have such a poor control apparatus in their sacral autonomic system.

*Episodic Diarrhoea*—some patients are subject to violent bouts of diarrhoea, often with vomiting, which last from twelve to forty-eight hours and which occur at intervals of one to six months. The disturbance may be akin to migraine or epilepsy—a sort of autonomic nerve storm. Nothing is known of the pathology or underlying mechanism. The sufferers are often nervous subjects prone to asthma, migraine or eczema.

